# Symptomatic and preventive effects of the novel phosphodiesterase-9 inhibitor BI 409306 in an immune-mediated model of neurodevelopmental disorders

**DOI:** 10.1038/s41386-021-01016-3

**Published:** 2021-05-03

**Authors:** Joseph Scarborough, Daniele Mattei, Cornelia Dorner-Ciossek, Michael Sand, Roberto Arban, Holger Rosenbrock, Juliet Richetto, Urs Meyer

**Affiliations:** 1 Institute of Pharmacology and Toxicology, University of Zürich-Vetsuisse, Zürich, Switzerland; 2 Department of CNS Discovery Research, Boehringer Ingelheim Pharma GmbH & Co KG, Biberach an der Riss, Germany; 3 Department of Medicine, Boehringer Ingelheim Pharmaceuticals Inc., Ridgefield, CT USA; 4 Neuroscience Center Zürich, University of Zürich and ETH Zürich, Zürich, Switzerland

**Keywords:** Pharmacology, Behavioural methods

## Abstract

BI 409306, a phosphodiesterase-9 inhibitor under development for treatment of schizophrenia and attenuated psychosis syndrome (APS), promotes synaptic plasticity and cognition. Here, we explored the effects of BI 409306 treatment in the polyriboinosinic-polyribocytidilic acid (poly[I:C])-based mouse model of maternal immune activation (MIA), which is relevant to schizophrenia and APS. In Study 1, adult offspring received BI 409306 0.2, 0.5, or 1 mg/kg or vehicle to establish an active dose. In Study 2, adult offspring received BI 409306 1 mg/kg and/or risperidone 0.025 mg/kg, risperidone 0.05 mg/kg, or vehicle, to evaluate BI 409306 as add-on to standard therapy for schizophrenia. In Study 3, offspring received BI 409306 1 mg/kg during adolescence only, or continually into adulthood to evaluate preventive effects of BI 409306. We found that BI 409306 significantly mitigated MIA-induced social interaction deficits and amphetamine-induced hyperlocomotion, but not prepulse inhibition impairments, in a dose-dependent manner (Study 1). Furthermore, BI 409306 1 mg/kg alone or in combination with risperidone 0.025 mg/kg significantly reversed social interaction deficits and attenuated amphetamine-induced hyperlocomotion in MIA offspring (Study 2). Finally, we revealed that BI 409306 1 mg/kg treatment restricted to adolescence prevented adult deficits in social interaction, whereas continued treatment into adulthood also significantly reduced amphetamine-induced hyperlocomotion (Study 3). Taken together, our findings suggest that symptomatic treatment with BI 409306 can restore social interaction deficits and dopaminergic dysfunctions in a MIA model of neurodevelopmental disruption, lending preclinical support to current clinical trials of BI 409306 in patients with schizophrenia. Moreover, BI 409306 given during adolescence has preventive effects on adult social interaction deficits in this model, supporting its use in people with APS.

## Introduction

Abnormal glutamatergic neurotransmission related to *N*-methyl-D-aspartate (NMDA) receptor hypofunction is implicated in the etiology of neuropsychiatric disorders, including schizophrenia [1–3]. NMDA receptors mediate Ca^2+^ entry into postsynaptic neurons, activating guanylyl cyclase via nitric oxide signaling to trigger postsynaptic production of cyclic guanosine monophosphate (cGMP), which acts in turn on a range of downstream protein targets to mediate synaptic plasticity [4, 5]. Signaling is terminated through cGMP hydrolysis mediated by phosphodiesterase (PDE) enzymes, particularly PDE9, which has higher affinity for cGMP than any other PDE isoform [5, 6].

BI 409306 is a novel PDE9 inhibitor, a class of compounds that are thought to promote NMDA receptor-related glutamatergic transmission by elevating postsynaptic levels of cGMP in neurons [4, 5]. In rodents, BI 409306 has been shown to increase cGMP in brain tissue and cerebrospinal fluid (CSF), promote synaptic plasticity (evaluated using hippocampal long-term potentiation), improve episodic memory, and reverse working memory deficits induced by acute pharmacological blockade of NMDA receptors [7]. Furthermore, dose-dependent increases in cGMP levels in the CSF of healthy volunteers have been observed after a single oral dose of BI 409306 [8]. Therefore, PDE9 inhibition with BI 409306 may provide benefits for patients with neurodevelopmental disorders by facilitating synaptic stabilization and plasticity-dependent NMDA receptor function. On this basis, ongoing clinical trials are investigating the potential of BI 409306 for the prevention of relapse in patients with schizophrenia treated with antipsychotic medications (NCT03351244), and for early intervention in patients with attenuated psychosis syndrome (APS) (NCT03230097).

Maternal immune activation (MIA) is an established experimental approach based on immune-mediated disruption of neurodevelopment in the offspring to induce brain and behavioral dysfunctions [9, 10]. Based on evidence highlighting a link between prenatal exposure to infectious or noninfectious MIA and neuropsychiatric disorders in the offspring [11], MIA approaches are commonly used to study the developmental trajectory of schizophrenia and other neurodevelopmental disorders [9–12]. In a commonly used MIA model, pregnant mouse dams are exposed to the viral mimic, polyriboinosinic–polyribocytidilic acid (poly[I:C]), a synthetic analog of double-stranded RNA that binds to transmembrane toll-like receptor 3, triggering an innate immune response [9]. Prenatal poly(I:C) treatment disrupts fetal development and induces lasting behavioral and cognitive abnormalities, including deficits in social behavior, sensorimotor gating, and dopaminergic neurotransmission in adult offspring [13–15]. MIA offspring display altered expression of the GluN1 subunit of NMDA receptors in the brain [16–18], altered basal extracellular glutamate levels, and modified responses to NMDA receptor antagonists [18–21]. These animals are therefore likely to have deficits in glutamatergic signaling that make them suitable for the investigation of compounds targeting this pathway.

We report the findings of three studies exploring the symptomatic and preventive effects of BI 409306 in the poly(I:C)-based MIA mouse model. Study 1 investigated the chronic effects of three doses of BI 409306 on MIA-induced behavioral deficits in adult offspring. These investigations aimed to evaluate the effect of PDE9 inhibition on MIA-induced behavioral deficits and to select an active dose for use in the subsequent studies. To examine the potential benefits of BI 409306 as an add-on to standard therapy (Study 2), BI 409306 was administered alone or in combination with risperidone, an antipsychotic drug acting primarily at dopamine D2 and serotonin 5-HT_2A_ receptors [22]. Finally, Study 3 investigated the ability of an active dose of BI 409306, administered during adolescence, to prevent the emergence of MIA-induced behavioral deficits in the adult offspring.

## Materials and methods

### Animals and ethical approval

Female and male C57Bl6/N breeder mice (10–12 weeks of age; Charles River Laboratories, Sulzfeld, Germany) were acclimatized in a temperature- and humidity-controlled (21 ± 3 °C, 50 ± 10%) specific-pathogen-free environment for 2 weeks under a reversed light–dark cycle (lights off: 09:00 a.m. to 09.00 p.m.), after which timed mating was conducted as previously described [23]. The animals were kept in individually ventilated cage systems (Tecniplast, Buguggiate, Italy) as previously described [24]. The cages were minimally enriched with a triangular plastic hut that allows only red-wavelength light to pass through (Tecniplast MOUSE HOUSE; Tecniplast, Buguggiate, Italy), wooden particle bedding, and additional nesting material provided by 2 sheets of regular paper tissues. All animals had ad libitum access to the same food (Kliba 3436, Kaiseraugst, Switzerland) and water throughout the entire study. All procedures were approved by the Cantonal Veterinarian’s Office of Zurich, and efforts were made to minimize the number of animals used.

### Drugs

Poly(I:C) potassium salt (lot number 117M4005V) and d-amphetamine sulfate (Amph) were obtained from Sigma-Aldrich (St Gallen, Switzerland). The molecular composition, purity, and immunopotency of the poly(I:C) lot used in this study has been previously evaluated [24]. Risperidone was obtained from Tocris Bioscience (Wiesbaden-Nordenstadt, Germany). BI 409306 was synthesized at Boehringer Ingelheim Italia, Chemistry Research Center (Milan, Italy). Pyrogen-free 0.9% NaCl solution (saline) was obtained from B. Braun (Melsungen, Switzerland).

### Breeding and maternal immune activation

The MIA model was implemented as previously reported [23–26] and full details are provided in Supplementary Table S1. In brief, successful mating was verified by the presence of a vaginal plug (designated as gestational day [GD] 0). Dams were housed individually throughout gestation. On GD 12, pregnant dams were randomly assigned to a single intravenous tail-vein injection of either poly(I:C) 5 mg/kg to induce MIA, or pyrogen-free 0.9% NaCl (prenatal control). All injections had a total volume of 5 mL/kg. Following the injection, dams were placed back in their home cages and left undisturbed until the first cage change on postnatal day (PND) 7. Offspring were weaned on PND 21 and littermates of the same sex were caged separately and maintained in groups of 4–5 animals per cage.

### Allocation and behavioral testing of offspring

Male and female offspring were allocated to daily treatment groups (Supplementary Table S2) and maintained as described above. All mice were subjected to a battery of behavioral tests in the following order, with 3–4 rest days between each test: (1) social interaction; (2) prepulse inhibition (PPI); (3) Amph-induced hyperlocomotion.

### BI 409306 and vehicle treatment

BI 409306 and/or risperidone were suspended in distilled water with 0.5% hydroxyethylcellulose and sonicated for 20 min, then mixed with 30% condensed milk solution at the required dose. For all treatments, 2 mL/kg of solution was administered using the micropipette-guided drug administration (MDA) method, as previously described [25]. On testing days, animals were treated 30 min before testing.

### Pharmacokinetic study

Male C57Bl6/N mice (10–12 weeks of age; Charles River Laboratories, Sulzfeld, Germany) were orally administered with BI 409306 0.5 mg/kg via MDA. Blood samples were taken via tail-vein sampling after 0.5, 1, and 2 h in 1.5 mL ethylenediaminetetraacetic acid (EDTA)-containing tubes and centrifuged (10,000 × *g*) for 10 min at 4 °C, as described previously [26]. The resulting plasma was stored at −20 °C. BI 409306 plasma concentrations were determined using liquid chromatography coupled to mass spectrometry, as previously described [25].

### Study 1: BI 409306 dose–response study in adult offspring

To limit the overall number of mice required to identify the optimal dose for subsequent studies, only male mice were used in the initial dose–response study. Male MIA offspring (7 litters; 36 mice) were assigned to daily BI 409306 0.2, 0.5, or 1 mg/kg, or vehicle control (Supplementary Table S2). Due to limited availability, prenatal control offspring (5 litters; 27 mice) were allocated to three treatment groups only (BI 409306 0.5 or 1 mg/kg, or vehicle control; Supplementary Table S2). Treatment began on PND 70 and continued throughout the experimental period, and behavioral testing began on PND 84.

One day after the final behavioral test, BI 409306 0.2, 0.5, or 1 mg/kg was administered orally to offspring using the MDA method in order to assess plasma exposure of the drug. Mice were sacrificed by decapitation 30 min later, and blood was collected from the trunk in 1.5 mL EDTA-containing tubes. Plasma concentrations of BI 409306 were determined as outlined above.

### Study 2: Study of add-on BI 409306 in combination with risperidone in adult offspring

Male and female MIA offspring (15 litters; 103 mice) were assigned to daily risperidone 0.025 or 0.05 mg/kg, risperidone 0.025 mg/kg plus BI 409306 1 mg/kg, BI 409306 1 mg/kg alone, or vehicle control (Supplementary Table S2). Risperidone doses were selected based on previous pilot experiments in the Amph-induced hyperlocomotion test. To evaluate the effect of MIA in the absence of drug treatment, male and female control offspring (5 litters; 23 mice) received daily treatment with vehicle control only. Treatment began on PND 77 and continued throughout the experimental period, and behavioral testing began on PND 91.

### Study 3: Prevention study in adolescent offspring

Male and female adolescent offspring from 14 MIA litters (65 mice; Supplementary Fig. S1 and Supplementary Table S2) and 15 prenatal control litters (64 mice; Supplementary Fig. S1 and Supplementary Table S2) were assigned to daily BI 409306 1 mg/kg (2 cohorts) or vehicle control (1 cohort) treatment starting at PND 30. After 4 weeks of treatment, one of the BI 409306-treated cohorts switched to daily treatment with vehicle control, while the other cohort continued treatment with BI 409306 1 mg/kg throughout the duration of behavioral testing (Supplementary Figure S1). Behavioral testing was performed between PND 74 and PND 100.

### Social interaction test

Social interaction was assessed using the relative time spent exploring an unfamiliar congenic mouse and an inanimate dummy object in a modified Y-maze, as described previously [26, 27]. During each trial, the mouse was allowed to explore the maze freely for 5 min. Social interaction was defined as nose contact within a 2 cm interaction zone. For each animal, a social preference index was calculated using the formula:

$$\left( \frac{{\left[ {\rm{time}}\;{\rm{spent}}\;{\rm{with}}\;{\rm{mouse}} \right]}}{{\left[ {\rm{time}}\;{\rm{spent}}\;{\rm{with}}\;{\rm{the}}\;{\rm{inanimate}}\;{\rm{object}} + {\rm{time}}\;{\rm{spent}}\;{\rm{with}}\;{\rm{the}}\;{\rm{mouse}} \right]}} \right) - 0.5$$

Positive social preference index values indicate a preference toward the unfamiliar mouse over the dummy object. Total distance moved during the test was also recorded, as a measure of general exploratory activity.

### PPI test

Sensorimotor processing was assessed using PPI of the acoustic startle reflex, as described previously [26, 28]. Animals were presented with a mixture of prepulse-only trials, pulse-only trials, prepulse–plus–pulse trials, and no stimulus trials. The prepulse and pulse were delivered by sudden elevation in broadband white noise level (lasting 40 and 20 ms, respectively) from background (65 dB_A_) with a rise time of 0.2–1.0 ms. Three pulse intensities (100, 110, and 120 dB_A_) and three prepulse intensities (71, 77, and 83 dB_A_, corresponding to +6, +12, and +18 dB_A_ above background) were used. PPI was calculated as percent inhibition of the startle response obtained in the pulse-alone trials using the formula:

$$100\% \times \left( {1 - \left[ {\frac{{\rm{mean}}\;{\rm{reactivity}}\;{\rm{in}}\;{\rm{prepulse}}\;{\rm{plus}}\;{\rm{pulse}}\;{\rm{trials}}}{{\rm{mean}}\;{\rm{reactivity}}\;{\rm{in}}\;{\rm{pulse}}\;{\rm{only}}\;{\rm{trials}}}} \right]} \right)$$

### Amphetamine-induced hyperlocomotion

Amph-induced hyperlocomotion was used to evaluate functional changes in dopaminergic neurotransmission, as previously described [27]. Animals explored the open field freely for 30 min before receiving an intraperitoneal injection of saline. Animals then explored the open field for a further 30 min before intraperitoneal administration of Amph 2.5 mg/kg (total volume 5 mL/kg). Locomotor responses were monitored for 90 min after Amph administration.

### Statistical analysis

The social interaction data were analyzed by analysis of variance (ANOVA), whereas the PPI and Amph-induced hyperlocomotion data were analyzed by repeated-measures ANOVA (RM-ANOVA), followed by Tukey’s post hoc test where appropriate. In the PPI test, prepulse and pulse intensities served as within-subject factors, whereas 5-min bins were used as within-subjects factors in the Amph-induced hyperlocomotion test. In the latter, the three phases of the test (baseline, saline, and Amph conditions) were analyzed separately.

The limited availability of prenatal control offspring in Study 1 precluded a full-factorial design with four treatment groups (BI 409306 0.2, 0.5, or 1 mg/kg, or vehicle control) in both prenatal treatment conditions. Hence, in Study 1, prenatal control offspring could only be assigned to three treatment groups (BI 409306 0.5 or 1 mg/kg, or vehicle control), whereas prenatal MIA offspring could be assigned to four MIA groups (BI 409306 0.2, 0.5, or 1 mg/kg, or vehicle control). As a consequence, a full-factorial two-way ANOVA on all groups was not feasible; therefore, the available data from Study 1 were analyzed using both one-way and two-way ANOVA or RM-ANOVA. Since the main objective of Study 1 was to perform a dose–response study in MIA offspring to identify the optimal dosage for our subsequent experiments (Study 2 and 3), we focused on the one-way ANOVAs or RM-ANOVAs in the analysis and in the main text of this article in order to present all treatment groups in the MIA offspring. The data from Study 1 that were analyzed by two-way ANOVA or RM-ANOVA are presented in the supplementary materials and aimed to evaluate possible main effects and interactions of treatments (BI 409306 0.5 or 1 mg/kg, or vehicle control) in both prenatal conditions. According to their experimental design, all data from Study 2 were analyzed by one-way ANOVA or RM-ANOVA, whereas data from Study 3 were analyzed by two-way ANOVA or RM-ANOVA. Preliminary analyses of the data from Studies 2 and 3 showed no sex-dependent effects, and so data from male and female offspring were combined to enhance statistical power. All statistical analyses were performed using SPSS Statistics (version 22.0; IBM, Armonk, NY, USA) and Prism (version 7.0; GraphPad Software, La Jolla, CA, USA). The threshold for statistical significance was set at *P* < 0.05.

## Results

### Pharmacokinetic study and plasma exposure

Following oral dosing with BI 409306 0.5 mg/kg via MDA [25], pharmacokinetic analysis in mice indicated that maximal plasma concentration of BI 409306 was reached within 30 min, with a mean (standard deviation [SD]) plasma concentration of 270 (65) nM (Supplementary Table S3). Based on the potency of BI 409306 against the PDE9 enzyme and a CSF/plasma ratio of 0.2 [7], this plasma exposure corresponds to a CSF exposure similar to the BI 409306 half maximal inhibitory concentration (IC_50_) against PDE9. Furthermore, this plasma exposure was in the range reported in clinical studies in healthy male volunteers and in patients with schizophrenia after a single oral dose of BI 409306 25 mg [8, 29].

### Study 1: BI 409306 dose–response study in adult offspring

**Fig. 1:**
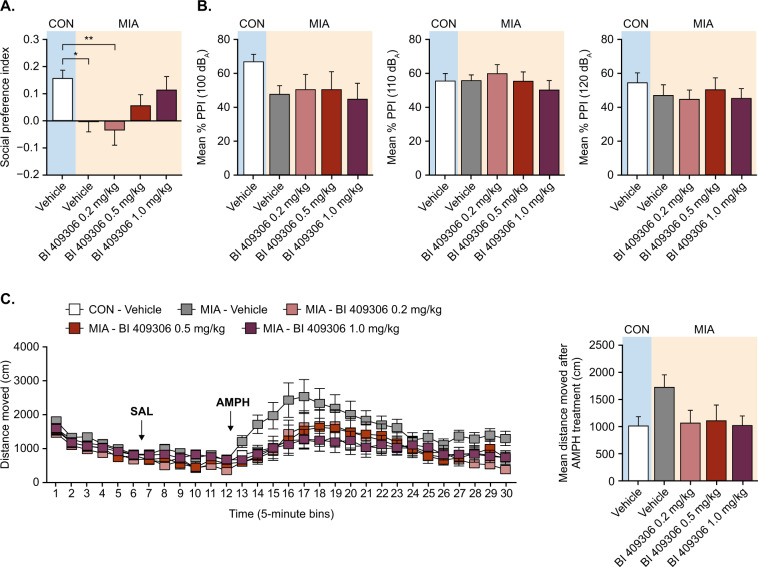
BI 409306 dose–response study in adult CON or MIA offspring (Study 1). (**A**) Effects of vehicle or drug exposure on social interaction, as indexed by the social preference index. (**B**) Effects of vehicle or drug exposure on PPI. (**C**) Effects of vehicle or drug exposure on Amph-induced hyperlocomotion in the open field. All values are means and error bars show the standard error of the mean. *N* = 8–10 mice per treatment group (POL animals originating from 7 litters, CON animals from 5 litters, respectively, for each treatment group). Data were analyzed using one-way ANOVA (social interaction test) or RM-ANOVA (PPI and Amph-induced hyperlocomotion tests) with post hoc Tukey’s tests. *P < 0.05; **P < 0.01 vs CON—Vehicle. Amph amphetamine, ANOVA analysis of variance, CON offspring of vehicle-treated control mice, MIA offspring of poly(I:C)-treated mice, PPI prepulse inhibition; SAL saline.

In the social interaction test, MIA offspring did not display a clear preference toward the live mouse, as indicated by a main effect of prenatal poly(I:C) treatment in the two-way ANOVA (*F*[1,48] = 7.332; *P* = 0.009; Supplementary Fig. S2), and by a main group effect in the one-way ANOVA (*F*[4,40] = 3.657; *P* = 0.013) showing a statistically significant difference between control offspring and MIA offspring in post hoc tests (*P* < 0.05; Fig. 1A). Treatment of MIA offspring with BI 409306 significantly reversed this deficit in a dose-dependent manner compared with vehicle control, with almost complete reversal observed at a dose of 1 mg/kg (Fig. 1A). These effects were not influenced by locomotor activity, as there were no group differences in total distance moved during the social interaction test (Supplementary Fig. S3).

A significant main effect of prenatal treatment on PPI was detected at the 100 dB_A_ pulse level, but not at higher pulse levels, leading to a significant interaction between pulse intensity and prenatal treatment in the two-way RM-ANOVA (*F*[2,96] = 3.44; *P* < 0.05). Subsequent two-way RM-ANOVA restricted to each pulse conditions confirmed a significant main effect of prenatal treatment in the 100 dB_A_ pulse condition (*F*[1,48] = 4.80; *P* < 0.01; Supplementary Fig. S4). Treatment with BI 409306 did not affect PPI, either when the data were analyzed by two-way RM-ANOVA (Supplementary Fig. S4) or by one-way RM-ANOVA (Fig. 1B).

Vehicle-treated MIA offspring displayed increased Amph-induced hyperlocomotion, which was mitigated by BI 409306 treatment as demonstrated by a significant interaction between prenatal treatment and drug treatment (two-way RM-ANOVA; *F*[2,48] = 3.40; *P* < 0.05; Supplementary Fig. 5). Comparison between CON and MIA groups using one-way RM-ANOVA failed to reach statistical significance, although BI 409306 treatment showed a trend for attenuation of distance moved following Amph treatment (main effect of groups; *F*[4,40] = 2.1; *P* = 0.09; Fig. 1C).

### Study 2: Add-on study of BI 409306 in combination with risperidone in adult offspring

**Fig. 2:**
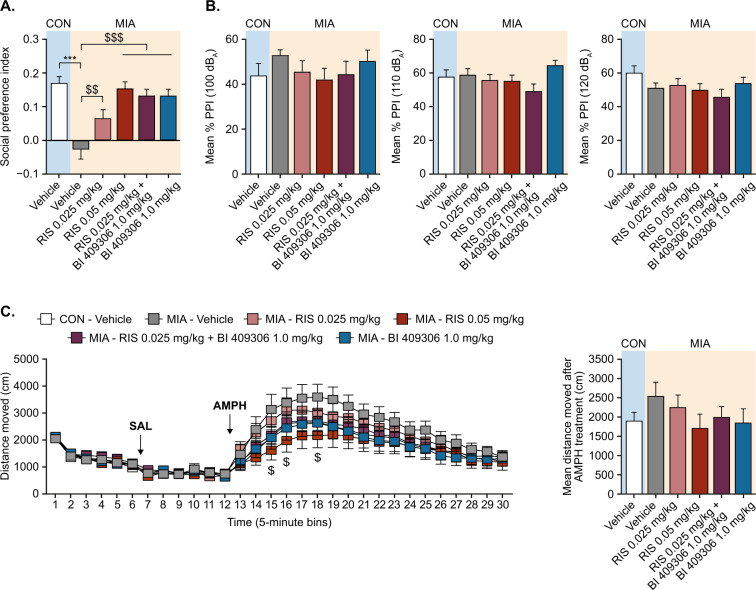
Study of add-on BI 409306 in combination with risperidone in adult CON or MIA offspring (Study 2). (**A**) Effects of vehicle or drug exposure on social interaction, as indexed by the social preference index. (**B**) Effects of vehicle or drug exposure on PPI. (**C**) Effects of vehicle or drug exposure on Amph-induced hyperlocomotion in the open field. All values are means and error bars show the standard error of the mean. *N* = 10–12 mice per sex per treatment group (each male and female animal in the MIA treatment groups is derived from a different litter; total MIA litters: 15, with 10–12 litters per group; CON animals from 5 litters). Data were analyzed using one-way ANOVA (social interaction test) or RM-ANOVA (PPI and Amph-induced hyperlocomotion tests) with post hoc Tukey’s tests. ***P < 0.001 vs CON— Vehicle, ^$^*P* < 0.05, ^$$^*P* < 0.01, ^$$$^P < 0.001 vs MIA—Vehicle. Amph amphetamine, ANOVA analysis of variance, CON offspring of vehicle-treated control mice, MIA offspring of poly(I:C)-treated mice, PPI prepulse inhibition, RIS risperidone; SAL saline.

In Study 2, social interaction differed significantly between treatment groups (main effect of treatment group *F*[5,120] = 11.83; *P* < 0.001; Fig. 2A); in post hoc tests, social interaction was significantly impaired in adult MIA offspring compared with control offspring (*P* < 0.001). Chronic daily treatment with BI 409306 1 mg/kg reversed this deficit when administered alone or in combination with risperidone 0.025 mg/kg (*P* < 0.001; Fig. 2A). Risperidone administered alone at 0.05 mg/kg also improved the social interaction deficits in MIA offspring (*P* < 0.001; Fig. 2A). Similar to Study 1 (Supplementary Fig. S3), there were no group differences in total distance moved (data not shown).

There were no significant group differences in PPI (Fig. 2B), suggesting that MIA failed to induce PPI deficits in this study. Moreover, treatment with BI 409306 alone or in combination with risperidone did not alter PPI in MIA offspring (Fig. 2B).

In the Amph-induced hyperlocomotion test, there was a significant interaction between treatment group and bins (*F*[85,2040] = 1.562; *P* = 0.001). Post hoc analyses of each 5-min bin revealed that Amph-induced hyperlocomotion in MIA offspring was significantly mitigated by risperidone 0.05 mg/kg. In addition, there was a nonsignificant attenuation by BI 409306, both alone and in combination with risperidone, compared with vehicle-treated MIA offspring (Fig. 2C).

### Study 3: Prevention study in adolescent offspring

**Fig. 3:**
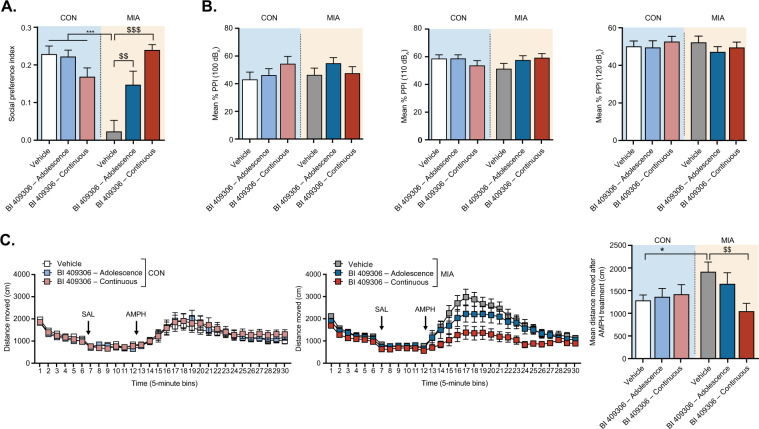
Effects of early chronic or preventive treatment with BI 409306 in adolescent CON or MIA offspring (Study 3). (**A**) Effects of vehicle or drug exposure on social interaction, as indexed by the social preference index. (**B**) Effects of vehicle or drug exposure on PPI. (**C**) Effects of vehicle or drug exposure on Amph-induced hyperlocomotion in the open field. All values are means and error bars show the standard error of the mean. *N* = 10–12 mice per sex per treatment group (each animal in every treatment group is derived from a different litter; total MIA litters: 14, with 10–12 litters per group; total CON litters:15, with 10–12 litters per group). Data were analyzed using two-way ANOVA (social interaction test) or RM-ANOVA (PPI and Amph-induced hyperlocomotion tests) with post hoc Tukey’s tests. *P < 0.05, ***P < 0.001 vs CON-Vehicle; ^$^*P* < 0.05, ^$$^*P* < 0.01, ^$$$^*P* < 0.001 vs MIA-Vehicle. Amph amphetamine; ANOVA analysis of variance, CON offspring of vehicle-treated control mice; MIA offspring of poly(I:C)-treated mice, PPI prepulse inhibition, SAL saline.

Consistent with the previous studies, MIA impaired social interaction, as evident from the substantial difference in social preference index between MIA offspring and control offspring treated with vehicle throughout the entire study (Fig. 3A). BI 409306 treatment, either in adolescence only or continuing throughout adulthood, prevented this deficit, as demonstrated by a significant interaction between prenatal treatment and drug treatment (*F*[2,123] = 14.21; *P* < 0.001; Fig. 3A). Post hoc analyses confirmed significant differences in the social preference index between MIA offspring treated with vehicle and those treated with BI 409306 1 mg/kg in adolescence only (*P* < 0.01) or throughout adulthood (*P* < 0.001; Fig. 3A). BI 409306 1 mg/kg did not affect social behavior in prenatal control offspring (Fig. 3A).

There were no significant group differences in PPI (Fig. 3B), suggesting that MIA failed to induce PPI deficits in this study. Likewise, BI 409306 treatment had no effect on PPI when administered either in adolescence only or throughout adolescence and adulthood (Fig. 3B).

Vehicle-treated MIA offspring displayed an increased sensitivity to the locomotor-stimulating effects of Amph, and this Amph hypersensitivity was prevented by BI 409306 when given continuously throughout adolescence and adulthood, but not by treatment during adolescence only (Fig. 3C). These outcomes were supported by the presence of a significant interaction between prenatal treatment and drug treatment (*F*[2,123] = 3.132; *P* < 0.05; Fig. 3C), and by post hoc analyses revealing a significant difference between vehicle-treated MIA offspring and MIA offspring treated continuously with BI 409306 1 mg/kg throughout adolescence and adulthood (*P* < 0.01; Fig. 3C).

## Discussion

This study explored the symptomatic and preventive effects of BI 409306 in offspring of the poly(I:C)-based MIA mouse model, which bears preclinical relevance for schizophrenia and other neurodevelopmental disorders [9, 11, 12]. In Study 1, social interaction deficits in adult male MIA offspring were significantly reversed, and Amph hypersensitivity was attenuated, by chronic daily oral treatment with BI 409306 1 mg/kg. Thus, this dose was selected as the active dose for use in the subsequent studies. In Study 2, chronic daily oral treatment of adult offspring with risperidone, BI 409306, or both drugs in combination reversed MIA-induced deficits in social interaction and ameliorated Amph hypersensitivity, whereas risperidone 0.05 mg/kg alone significantly reversed the locomotor response to Amph. In Study 3, chronic daily oral treatment with BI 409306 in adolescence only was sufficient to significantly prevent the social interaction deficit in MIA offspring, but did not mitigate the increased Amph sensitivity. However, treatment with BI 409306 starting in adolescence and continuing throughout adulthood prevented both MIA-induced social interaction deficits and dopaminergic abnormalities assessed by the Amph hyperlocomotion test.

Our study did not provide any evidence for sex-dependent effects of MIA and/or BI 409306 treatment. In keeping with the fact that maternal poly(I:C) administration was conducted on GD 12, the lack of sex-dependent MIA effects is consistent with our previous findings, showing that poly(I:C)-induced MIA in early or middle gestation (GD 9 or 12) mostly failed to induce robust sex-specific effects on behavior [24, 26, 30], whereas identical MIA at a later gestational time period (e.g., GD 17) is associated with remarkable sex-specific effects [31]. Hence, the precise prenatal timing appears to be one of the factors determining the extent to which poly(I:C)-induced MIA produces sex-dependent or -independent effects on behavior. The sex-independent effects of the pharmacological treatments (BI 409306 alone, risperidone alone, or BI 409306 plus risperidone) reported here are consistent with our previous investigations, which revealed comparable behavioral effects of chronic (4 weeks) haloperidol, clozapine, or fluoxetine administration in adolescent male and female mice that were exposed to poly(I:C)-induced MIA [32]. Similar sex-independent effects were also obtained in other mouse models of poly(I:C)-induced MIA, which assessed the behavioral and cognitive effects of chronic (2 weeks) treatment with haloperidol or clozapine in adulthood [14]. Against this background, our findings suggest that pharmacological inhibition of PDE9 is effective in both male and female mice, at least with regards to mitigating social interaction deficits and hypersensitivity to amphetamine.

A deficit in PPI was found in MIA offspring in Study 1, but this deficit was not ameliorated by BI 409306, suggesting that pharmacological inhibition of PDE9 may not be effective in mitigating MIA-induced deficits in PPI. This notion is consistent with a previous preclinical study of the PDE9 inhibitor PF-4447943, which showed no effect on PPI in naive mice when administered alone [33]. In our study, BI 409306 also had no effect on PPI when combined with risperidone, which contrasts with previous findings that PF-4447943 in combination with risperidone improves PPI in naive mice [33]. A possible reason for this discrepancy may relate to differences in baseline levels of PPI. In the study of Kleiman et al. [33], vehicle-treated control mice (C57BL6/J background) displayed mean PPI levels of less than 30%, whereas the baseline PPI levels in our vehicle-treated control mice (C57BL6/N background) were higher (40–60% on average). While it has been shown that pharmacological compounds such as antipsychotics can increase PPI in mice that exhibit inherently low levels of PPI [34], the relatively high baseline PPI levels in our study may have masked a possible effect of BI 409306 alone or in combination with risperidone on PPI due to ceiling effects. The latter may also provide a parsimonious explanation as to why we failed to reveal an effect of risperidone alone on PPI here (Study 2) and in previous studies [25]. Moreover, because poly(I:C)-induced MIA was found to induce only subtle effects on PPI in the present study, possible ceiling effects may have precluded the identification of BI 409306 treatment effects in MIA offspring.

MIA offspring display altered glutamatergic signaling related to NMDA receptor hypofunction, which originates during development and is thought to underlie some of the behavioral and cognitive deficits seen in these animals [16, 19]. While also having relevance for other neurodevelopmental disorders, some of MIA-induced behavioral and cognitive abnormalities are reminiscent of and relevant to the symptoms of schizophrenia and related psychotic disorders [9, 11, 12]. Our finding that BI 409306 treatment in adulthood can ameliorate MIA-induced social deficits and dopaminergic dysfunction may, therefore, indicate a potential symptomatic benefit for adult patients with schizophrenia. These preclinical data lend additional support to the rationale for the ongoing Phase II trial of BI 409306 as an add-on therapy for the prevention of relapse in adult patients with schizophrenia (NCT03351244). In the present study, the observed plasma exposure of BI 409306 1 mg/kg was in the range that would be expected for human participants after a 50 mg dose, based on exposure levels observed in previous clinical studies in healthy volunteers and patients with schizophrenia [7, 8, 29]. This dose is used in the ongoing clinical trials on prevention of relapse in schizophrenia [10] and early intervention in patients with APS [35], further supporting the relevance of our findings to patients.

BI 409306 reversed MIA-induced social interaction deficits in adulthood in a similar manner when administered alone or in combination with risperidone, an established treatment for psychosis [22]. Similar to other medications currently used to control symptoms of psychosis, risperidone acts predominantly on the dopaminergic and serotoninergic signaling pathways, blocking D2 and 5-HT_2A_ receptors among others [22]. In contrast, BI 409306 is thought to act primarily on NMDA receptor-mediated glutamatergic signaling via the nitric oxide/cGMP pathway, leading to improved synaptic plasticity [5, 7]. Given that BI 409306 and risperidone act on distinct targets and neurotransmitter systems, the combination of these two treatments may have resulted in further improvements that could not be detected in the social interaction and Amph-induced hyperlocomotion tasks due to ceiling effects. This will be explored further in the ongoing Phase II trial (NCT03351244), which will determine the benefit of BI 409306 as add-on therapy to standard care and establish whether combining treatments that act on different molecular targets is advantageous for the prevention of relapse in patients with schizophrenia [10].

The neurodevelopmental origin of schizophrenia is thought to involve deficits in the maturation of glutamatergic and γ-amino butyric acid (GABA)ergic networks in the prefrontal cortex [36–38]. During early brain development, changes in the glutamatergic and GABAergic signaling result in alterations to the properties of the excitatory/inhibitory network that are required for normal cognitive function [39–42]. These processes represent a critical period in neurodevelopment, during which environmental insults may disrupt the maturation of prefrontal signaling networks and increase the risk of developing long-term social and cognitive deficits [39–42]. In the present study, BI 409306 treatment during adolescence was sufficient to prevent the emergence of social interaction deficits, whereas continuation of treatment into adulthood was necessary to also prevent dopaminergic dysfunction in MIA-exposed offspring. Therefore, treatments targeting glutamatergic transmission, such as BI 409306, during critical periods of development may have a preventative mechanism of action and improve long-term outcomes for patients at high risk of developing schizophrenia. This supports the rationale for an ongoing trial of BI 409306 as an early intervention for patients with APS [35].

Certain limitations should be considered in the interpretation of our findings. First, we observed some variability in the effects of MIA between studies, especially with regard to its effects on PPI. Indeed, MIA-induced deficits in PPI were observed in Study 1 but were not present in subsequent studies. Consistent with the existing data [43], our experience suggests that PPI is more prone to variable outcomes in the poly(I:C)-based mouse model of MIA than social interaction, which was another behavioral readout included in the present study. We have recently explored behavioral variability in the poly(I:C)-based MIA model by comparing the contribution of within- and between-litter variation in a cohort of >150 MIA and control mice [26]. In that study, we used unsupervised cluster analyses to identify subgroups of MIA offspring with differing behavioral profiles. We identified that ~40% of mice do not display overt deficits in PPI, and this variability primarily stemmed from within-litter rather than between-litter variation [26]. Even though the presence or absence of social interaction deficits in adult MIA-exposed mice was predictive of the presence or absence of PPI deficits when subgroups were taken into account [26], the correlation between these two measures is rather weak at the level of the individual offspring (unpublished observation). Hence, an individual MIA-exposed mouse offspring may display a strong deficit in social interaction, but only minimal PPI impairments. These findings suggest that the magnitude of MIA-induced changes in behavior depends on the precise behavioral or cognitive measure, and consequently it is challenging to run prior power analyses to detect a behavioral effect of MIA in the PPI paradigm when multiple readouts (e.g., social interaction, PPI and amphetamine sensitivity) are simultaneously assessed in the same study. At this stage, we have no evidence-based explanation as to why PPI is more prone to variable outcomes in the poly(I:C)-based mouse model of MIA compared with other behavioral readouts such as social behavior, but its variability may be influenced by numerous factors, including the precise prenatal timing [18], intensity of the maternal immune response [23], and varying exposure to internal (e.g., social hierarchies) as well as external (e.g., experimental stimuli or manipulations) factors prior to testing [44]. Nonetheless, MIA produced robust deficits in social behavior and dopamine-related functional anomalies (assessed using Amph-induced hyperlocomotion) in adult offspring across the three studies.

Another limitation of our study is that its experimental design precluded a discrimination of short-term (acute) and long-term (chronic) effects of BI 409306 treatment on behavior. Our study was primarily designed as a preclinical investigation matching, as far as possible, the ongoing clinical trials with BI 409306 [10, 35], in which the enrolled participants are evaluated and characterized under continuous treatment. Future studies assessing BI 409306 treatment in preclinical models should include experimental designs in which possible short-term (acute) and long-term (chronic) effects of BI 409306 treatment could be evaluated separately. Finally, it should be noted that the MIA model, like any other model, does not capture all aspects of schizophrenia, but instead reflects certain key features of the behavioral phenotypes and neurodevelopmental pathology associated with the disorder [45].

In conclusion, we present the first studies to use a MIA-based neurodevelopmental disruption model to demonstrate the efficacy of a novel drug in ameliorating behavioral deficits related to glutamatergic neurotransmission. Taken together, our findings demonstrate that chronic treatment with BI 409306 in adolescence or adulthood can lead to attenuation or prevention, respectively, of social interaction deficits and dopaminergic dysfunction in a neurodevelopmental mouse model with relevance to schizophrenia. In addition, BI 409306 treatment could provide improvements alone and in combination with established treatments for psychosis acting on the dopaminergic and serotoninergic systems. These novel data support the ongoing development of BI 409306 for the prevention of relapse in patients with schizophrenia [10] and as an early intervention in patients with APS [35].

## Funding and disclosure

This study was funded by Boehringer Ingelheim Pharma GmbH & Co KG. The sponsor was given the opportunity to review the manuscript for medical and scientific accuracy as well as intellectual property considerations. JS, DM, and JR have no conflicts of interest to declare. CD-C, MS, RA, and HR are employees of Boehringer Ingelheim, but received no direct compensation related to the development of this manuscript. UM has received financial support unrelated to the present study from Wren Therapeutics Ltd. Open Access funding provided by Universität Zürich.

## Supplementary information

Supplement

Supplementary Table 1
