# Supplementary material for: Symptomatic and preventive effects of the novel phosphodiesterase-9 inhibitor BI 409306 in an immune-mediated model of neurodevelopmental disorders
Source: Neuropsychopharmacology. 2021 May 3;46(8):1526–34. doi: 10.1038/s41386-021-01016-3 (PMC8209175; doi:10.1038/s41386-021-01016-3)
Supplement: Supplementary file 1 — Supplement [file 41386_2021_1016_MOESM1_ESM.pdf]

Supplementary Tables

Supplementary Table S1 Full details of MIA model

ARRIVE, Animal Research: Reporting of In vivo Experiments; IVC, individually ventilated cage; MIA, maternal immune activation; PND, postnatal day; RT, room temperature

Supplementary Table S2. Number of offspring included in each study and group.

Study	Experimental Group	M	F	Total
1	CON-Vehicle	9 (5)	-	9
	CON-BI 409306 0.5 mg/kg	9 (5)	-	9
	CON-BI 409306 1 mg/kg	9 (5)	-	9
	MIA-Vehicle	10 (7)	-	10
	MIA-BI 409306 0.2 mg/kg	9 (7)	-	9
	MIA-BI 409306 0.5 mg/kg	8 (7)	-	8
	MIA-BI 409306 1 mg/kg	9 (7)	-	9
				**63**
2	CON-Veh	11 (5)	12 (5)	23
	MIA-Veh	11 (11)	10 (10)	21
	MIA-RIS 0.025 mg/kg	10 (10)	12 (12)	22
	MIA-RIS 0.025 mg/kg + BI 409306 1 mg/kg	10 (10)	10 (10)	20
	MIA-RIS 0.05 mg/kg	10 (10)	10 (10)	20
	MIA-BI409306 1 mg/kg	10 (10)	10 (10)	20
				**126**
3	CON-Vehicle	11 (11)	10 (10)	21
	CON-BI 409306 1 mg/kg adolescence	10 (10)	12 (12)	22
	CON-BI 409306 1 mg/kg continuous	11 (11)	10 (10)	21
	MIA-Vehicle	10 (10)	10 (10)	20
	MIA-BI 409306 1 mg/kg adolescence	12 (12)	12 (12)	24
	MIA-BI 409306 1 mg/kg continuous	11 (11)	10 (10)	21
				**129**

CON, control offspring; MIA, offspring of poly(I :C)-treated mice; RIS, risperidone. In brackets: number of litters originating the animals included in each experimental group.

Supplementary Table S3. Plasma concentrations of BI 409306 in mice 0.5, 1, and 2 hours after oral administration of BI 409306 0.5 mg/kg via MDA.

Time, h	Mean (SD) plasma concentration, nM
0.5	270 (65)
1.0	158 (41)
2.0	37 (21)

N=3 animals

MDA, micropipette-guided drug administration; SD, standard deviation

Supplementary Figures

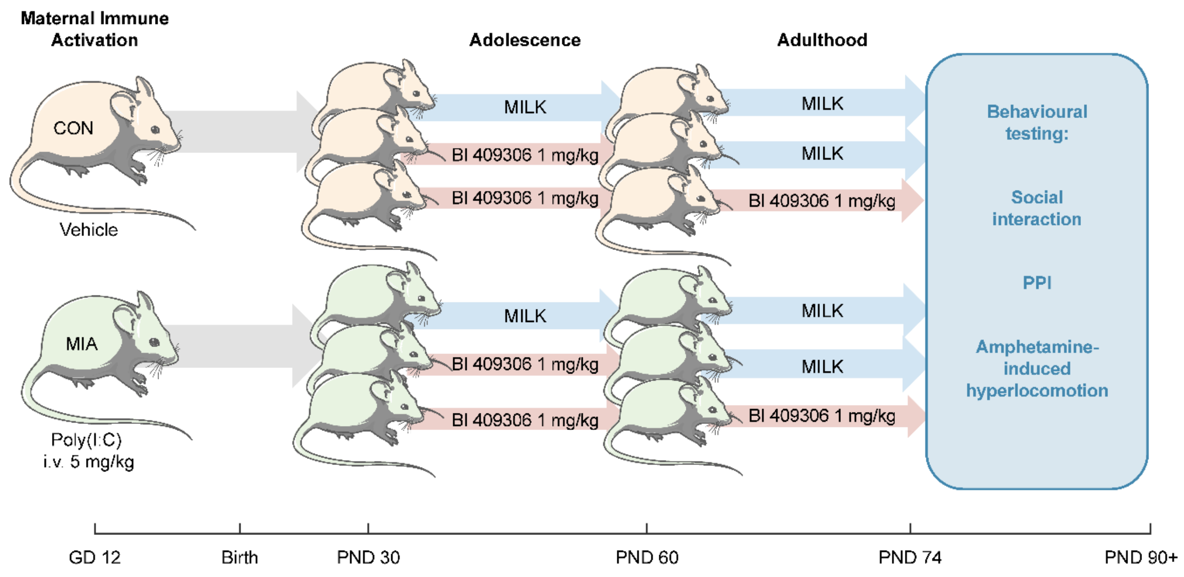

Supplementary Figure S1. Schematic representation of the experimental design of Study 3. Male and female offspring of vehicle-treated (CON) or poly(I:C)-treated (MIA) mothers were assigned to daily BI 409306 1 mg/kg (2 cohorts) or vehicle control (1 cohort) treatment starting at PND 30. After 4 weeks of peri-adolescent treatment, one of the BI 409306-treated cohorts switched to daily treatment with vehicle control, while the other cohort continued treatment with BI 409306 1 mg/kg throughout the duration of behavioral testing. Behavioral testing was performed between PND 72 and PND 100.

CON, vehicle-treated control mice; GD, gestational day; i.v., intravenous; MIA, poly(I:C)-treated mice; PND, postnatal day; PPI, prepulse inhibition

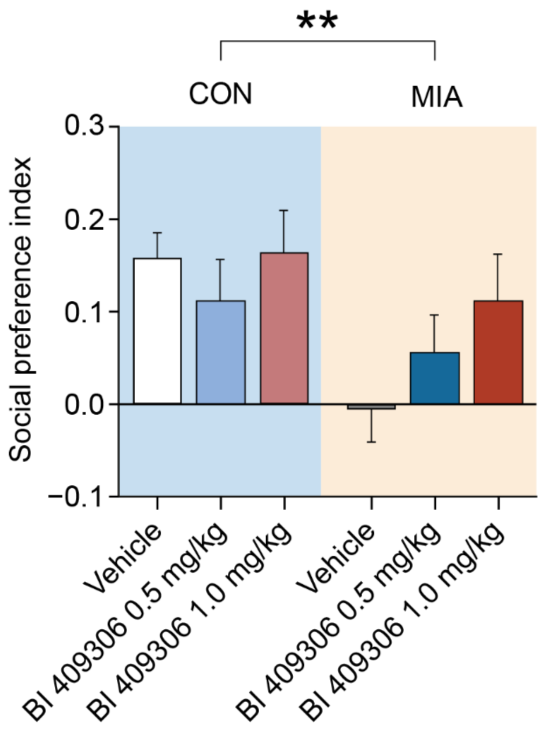

Supplementary Figure S2. Effects of MIA in Study 1 on social interaction as analyzed using a full-factorial design to evaluate possible main effects and interactions of BI 409306 treatments in both prenatal conditions. All values are means and error bars show standard error of the mean. N=8–10 mice per treatment group. Data were analyzed using 2-way ANOVAs with post hoc Tukey's tests. **P<0.01 for main effect of MIA.

ANOVA, analysis of variance; CON, offspring of vehicle-treated control mice; MIA, offspring of poly(I:C)-treated mice; PPI, prepulse inhibition

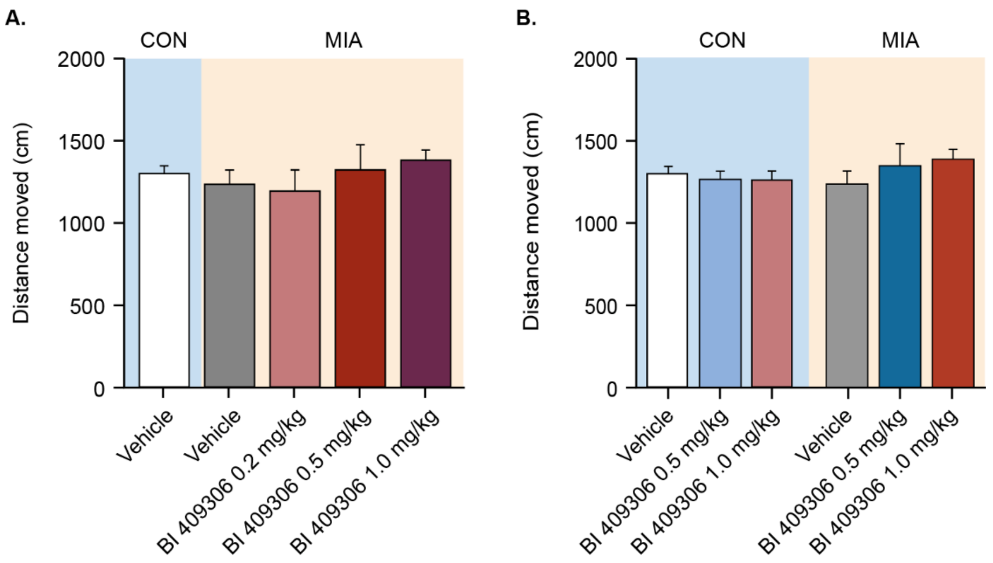

Supplementary Figure S3. Locomotor activity scores in the social interaction test as analyzed using (A) 1-way ANOVA and (B) 2-way ANOVA. All values are means and error bars show standard error of the mean. N=8–10 male mice per treatment group.

ANOVA, analysis of variance; CON, offspring of vehicle-treated control mice; MIA, offspring of poly(I:C)-treated mice

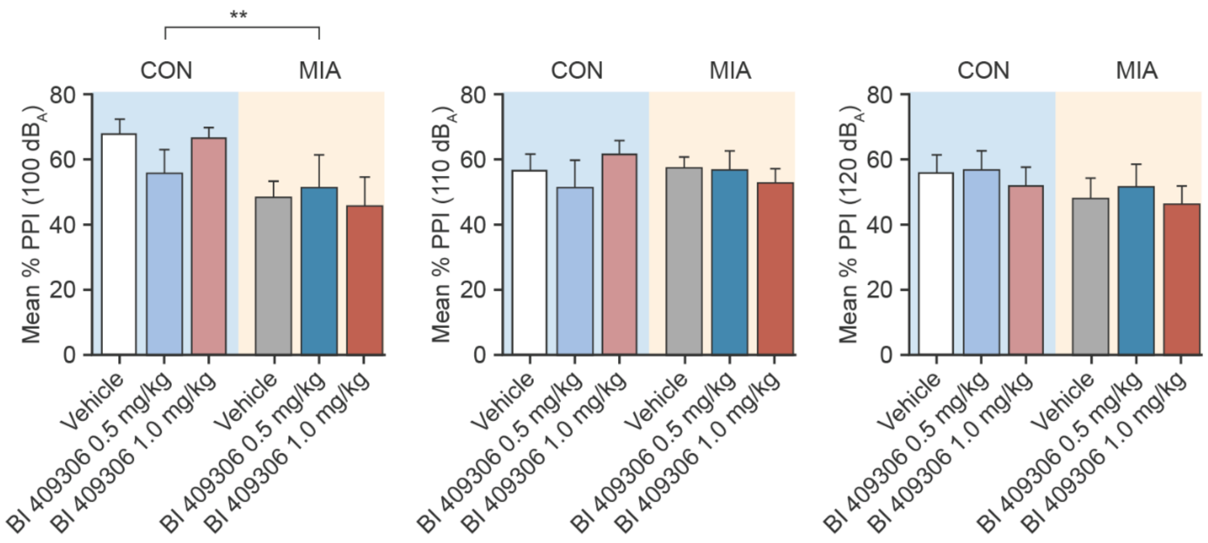

Supplementary Figure S4. Effects of MIA in Study 1 on prepulse inhibition as analyzed using a full-factorial design to evaluate possible main effects and interactions of BI 409306 treatments in both prenatal conditions. The bar plots show mean % PPI values for each of the three pulse conditions (pulse A, B, and C, which correspond to 100, 110, and 120 dB_A). All values are means and error bars show standard error of the mean. N=8–10 mice per treatment group. Data were analyzed using 2-way ANOVAs with post hoc Tukey's tests. **P<0.01 for main effect of MIA.

ANOVA, analysis of variance; CON, offspring of vehicle-treated control mice; MIA, offspring of poly(I:C)-treated mice; PPI, prepulse inhibition

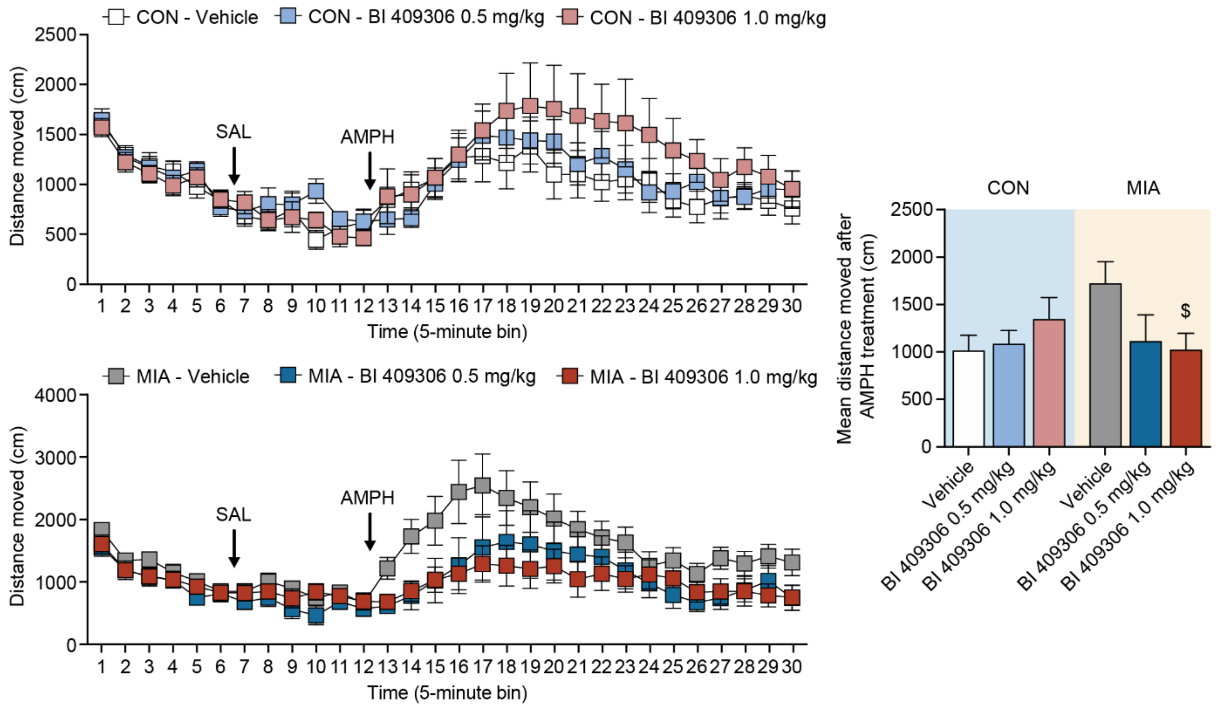

Supplementary Figure S5. Effects of MIA in Study 1 on Amph sensitivity as analyzed using a full-factorial design to evaluate possible main effects and interactions of BI 409306 treatments in both prenatal conditions. Line plots show distance moved as a function of 5-minute bins during the initial habituation phase (bins 1–6), saline treatment phase (bins 7–12) and AMPH treatment phase (bins 13–30). The bar plot shows the mean distance moved during the entire AMPH treatment phase. All values are means and error bars show standard error of the mean. N=8–10 mice per treatment group. Data were analyzed using 2-way ANOVAs with post hoc Tukey's tests. $^{\$}P<0.05$ vs MIA – Vehicle.

Amph, amphetamine; ANOVA, analysis of variance; CON, offspring of vehicle-treated control mice; MIA, offspring of poly(I:C)-treated mice; PPI, prepulse inhibition
